# A Prognostic Score for the Prediction of Local Treatment Failure in Plaque Brachytherapy of Uveal Melanoma

**DOI:** 10.1016/j.adro.2022.101152

**Published:** 2022-12-25

**Authors:** Ruba Kal Omar, Anna Hagström, Simon Dahlander, Åsa Carlsson Tedgren, Gustav Stålhammar

**Affiliations:** a Department of Medicine, Karolinska Institutet, Stockholm, Sweden; b Department of Clinical Neuroscience, Division of Eye and Vision, Unit of Ocular Oncology and Pathology, St. Erik Eye Hospital, Karolinska Institutet, Stockholm, Sweden; c Medical Radiation Physics and Nuclear Medicine, Karolinska University Hospital, Stockholm, Sweden; d Department of Oncology and Pathology, Karolinska Institutet, Stockholm, Sweden; e Radiation Physics, Department of Medical and Health Sciences, Linköping University, Linköping, Sweden; f St. Erik Eye Hospital, Stockholm, Sweden

## Abstract

**Purpose:**

To develop a prognostic score that correlates to a low, medium, and high incidence of treatment failure after plaque brachytherapy of uveal melanoma (UM).

**Methods and Materials:**

All patients who have received plaque brachytherapy for posterior UM at St. Erik Eye Hospital in Stockholm, Sweden from 1995 through 2019 were included (n = 1636). Treatment failure was defined as tumor recurrence, lack of tumor regression, or any other condition requiring a secondary transpupillary thermotherapy (TTT), plaque brachytherapy, or enucleation. The total sample was randomized into 1 training and 1 validation cohort, and a prognostic score for the risk for treatment failure was developed.

**Results:**

In multivariate Cox regression, low visual acuity, tumor distance to the optic disc ≤2 mm, American Joint Committee on Cancer (AJCC) stage, and a tumor apical thickness of >4 (for Ruthenium-106) or >9 mm (for Iodine-125) were independent predictors of treatment failure. No reliable threshold could be identified for tumor diameter or cancer stage. In competing risk analyses of the validation cohort, the cumulative incidence of treatment failure, as well as of secondary enucleation, increased with the prognostic score: In the low, intermediate, and high-risk classes, the 10-year incidence of treatment failure was 19, 28, and 35% and of secondary enucleation 7, 19, and 25 %, respectively.

**Conclusions:**

Low visual acuity, American Joint Committee on Cancer stage, tumor thickness, and tumor distance to the optic disc are independent predictors of treatment failure after plaque brachytherapy for UM. A prognostic score was devised that identifies low, medium, and high risk for treatment failure.

## Introduction

Uveal melanoma (UM) is the most common primary intraocular malignancy in adults, with a 20-year relative survival rate of about 60%.^1^ Episcleral plaque brachytherapy is the first-line treatment for most primary tumors.^2^^,^^3^ In the large, randomized Collaborative Ocular Melanoma Study (COMS), the 5-year Kaplan-Meier treatment failure rate after plaque brachytherapy was 10%, defined as tumor growth by ≥15% upon echography or a 250-μm expansion of any tumor boundary as judged from photographs or clinical examination on 2 subsequent occasions.^4^ In other studies, the definitions and incidences of tumor recurrences have varied considerably; Damato et al reported a 5-year tumor recurrence rate of 2%, defined as unequivocal expansion of any tumor margin as determined by the examining ophthalmologist, whereas Barker et al observed a 42% recurrence rate, defined according to the COMS criteria.^5^^,^^6^ However, tumor recurrence is not the only possible type of treatment failure. Lack of tumor regression after treatment, scleral necrosis, tumor lysis with uncontrollable uveitis, severe radiation neuropathy or retinopathy, or a blind painful eye that requires enucleation, a second round of plaque brachytherapy, or transpupillary thermotherapy (TTT) may all be considered failures of the initial treatment.^7^, ^8^, ^9^, ^10^ Any such event can cause distress, discomfort, and loss of vision for patients while consuming significant resources from healthcare systems. Further, in a collaborative study from 2016, tumor recurrence after plaque brachytherapy was associated with significantly shorter metastasis-free survival.^11^

Reducing the rates of treatment failure of UM is therefore an important goal for ocular oncologists. Several studies have investigated risk factors that may predict treatment failure, including greater tumor dimensions, retinal detachment, advanced patient age, young patient age, tumor proximity to the optic disc and fovea, and low baseline visual acuity.^4^, ^5^, ^6^^,^^12^, ^13^, ^14^, ^15^, ^16^, ^17^ Conversely, absorbed dose and dose rate at the tumor apex within reasonable limits are not strong risk factors for treatment failure, where we have previously shown that the absorbed dose (between 73 and 109 Gy) as well as the dose rate (between 0.5 and 2.8 Gy/hr) at the tumor apex are not associated with ocular or patient survival.^8^^,^^9^ Some clinicians use a minimal scleral dose of 300 to 700 Gy to ensure sufficient treatment margins and enable the development of choroidal atrophy that can be used to verify correct plaque positioning.^5^^,^^18^^,^^19^ A study by Espensen et al found that a minimum tumor dose of 100 Gy with Ruthenium-106 (Ru-106), the radioisotope used mostly in Europe for brachytherapy, was required to achieve ≥84% local tumor control at 3 years, while Iodine-125 (I-125), another radioisotope, has been established as the gold standard when treating medium-sized UM.^20^^,^^4^ None of these aforementioned studies, however, have provided a framework that guides clinicians and patients outside the analyzed data sets regarding their risk for treatment failure. If this risk is unacceptably high, one might consider enucleation instead of plaque brachytherapy. Further, most analyses of recurrence or treatment failure rates have relied on actuarial methods that may overstate rates in presence of competing risks (ie, patient death). Herein, we aimed to develop and validate a treatment failure prognostic score that correlates with the cumulative incidence of treatment failure and may aid clinicians and patients in determining an appropriate treatment alternative.

## Methods and Materials

The study adhered to the tenets of the Declaration of Helsinki, and the protocol was approved by the Swedish Ethical Review Authority (reference 2022-00930-02). All patients who received plaque brachytherapy for posterior UM (in the choroid and/or ciliary body. Iris melanomas excluded) at St. Erik Eye Hospital, in Stockholm, Sweden, from January 1, 1995, through January 1, 2020, were considered for the study (n = 1935). Retrospective data were retrieved from the digitalized Brachytherapy of Uveal Melanoma treatment directory, including data on patient age and sex, tumor size and location, radioisotope (Ru-106 or I-125), retreatment with secondary enucleation, secondary plaque brachytherapy, TTT, and patient survival.

All patients received their diagnosis at St. Erik Eye Hospital based on slit-lamp biomicroscopy and A- and B-scan ultrasonography. Fluorescein angiography, optical coherence tomography, wide-field fundus photographs, and radiologic examinations could be added at the discretion of the attending ophthalmologist. Patients were asked about previous diseases, medications, and family history and gave personal descriptions of their symptoms and symptom duration. The ophthalmologist then recorded the symptoms as 1 or several of (1) a shadow in the visual field, (2) photopsia or floaters, (3) metamorphopsia, (4) ocular pain, or (5) a combination of these. Plaque brachytherapy was typically performed within 4 weeks after diagnosis.

In review of the data in the Brachytherapy of Uveal Melanoma treatment directory, 299 patients were excluded because of primary enucleation treatment despite an initial evaluation for plaque brachytherapy. Of these patients, 278 had tumors that were deemed to be too large or at a location that was suboptimal for satisfactory plaque placement. Twenty patients had a strong preference for enucleation. One patient's diagnosis was changed to metastatic breast carcinoma after transvitreal biopsy. Excluding the above patients, 1636 patients remained for analysis.

Our protocols for Ru-106 and I-125 plaque brachytherapy, available plaque types, dosimetry calculations, and verification of source specification data have been described previously.^8^^,^^9^^,^^21^

Regular follow-up of all patients was scheduled at 1, 3, 6, and 12 months after therapy and annually or semiannually thereafter if local control had been achieved. Semiannual screening for liver metastases by ultrasonography or computed tomography was performed for 5 years after diagnosis, and thereafter only when prompted by symptoms, palpable masses, deteriorating health, jaundice, weight loss, or other signs.

### Statistical analyses

*P* values <0.05 were considered statistically significant, with all *P* values being 2-sided. Pretreatment visual acuity was measured on a Monoyer-Granström decimal scale chart at a distance of 5 m ranging from 2.0 (corresponding to 20/10) to 0. Modified from standards in the Swedish National Quality Registry for Cataracts, counting fingers at a distance of 4 m was recorded as 0.08, hand movements as 0.04, perception and localization (L + P) of light as 0.01, and amaurosis as 0. Average visual acuities were calculated on -1 LogMar converted values. Visual impairment was defined according to the International Classification of Diseases by the World Health Organization, in which distance vision <6/12 to 6/18 (corresponding to LogMAR ≥0.30) is classified as mild impairment or worse.^22^ Treatment failure was defined as tumor recurrence, lack of tumor regression, scleral necrosis, tumor lysis with uncontrollable uveitis, severe radiation neuropathy or retinopathy, or a blind painful eye that required a secondary TTT, plaque brachytherapy, or enucleation. Reduced visual acuity, discomfort, cataract, macular edema, vitreous bleedings, glaucoma, or transient uveitis that did not require any of these interventions were not classified as treatment failure. The competing event in cumulative incidence analyses was all-cause mortality, defined as death from any reason. Uni- and multivariate Cox regressions were used for analyses of hazard ratios (HRs) for treatment failure. To avoid overfitting the prognostic score to our own data, the patient sample was randomized into 1 training and 1 validation cohort in a 1:1 ratio with the RANDBETWEEN function in Excel for Mac (version 16.60, Microsoft Corporation, Redmond, Washington, USA). Thresholds for classification were obtained with receiver operating characteristics. For each factor, the threshold associated with highest accuracy (sensitivity plus specificity) for treatment failure was selected for inclusion in the final prognostic score, which was then tested in the validation cohort. Cumulative incidences of treatment failure were plotted from competing risk data with the *cmprsk* package for R, and the equality of incidence distributions was tested with Gray's test for equality.^23^ All other statistical analyses were performed using IBM SPSS statistics (version 27, Armonk, New York, USA) and GraphPad Prism (version 9.3.0, San Diego, California, USA).

## Results

### Descriptive statistics

Of the 1636 included patients, 813 (50%) were women and 823 (50%) were men. Their mean age at diagnosis was 64 years (standard deviation, 14). Only 3 patients (<1%) had tumors with extrascleral extension, typically an indication for enucleation. Two patients (<1%) had metastatic disease at the time of plaque brachytherapy. Five hundred eighty-five patients died during follow-up, of which 369 died of metastatic UM. The median follow-up for the 1051 survivors was 7.2 years (interquartile range, 9.8; Table 1).

**Table 1:** Demographics and clinical features of study patients

Feature	Distribution
n	1636
Age at diagnosis, mean (SD)	64 (14)
Sex, n (%)	
Female	813 (50)
Male	823 (50)
Visual acuity at diagnosis, mean LogMAR (SD)	0.32 (0.45)
Tumor thickness at diagnosis, mean mm (SD)	5.2 (2.5)
Tumor diameter at diagnosis, mean mm (SD)	10.6 (3.6)
Ciliary body involvement, n (%)	
Yes	88 (5)
No	1548 (95)
Extrascleral extension, n (%)	
Yes	3 (<1)
No	1633 (100)
AJCC stage at diagnosis, n (%)	
I	536 (33)
IIA	710 (43)
IIB	250 (15)
IIIA	44 (3)
IIIB	0 (0)
IIIC	0 (0)
IV	2 (<1)
N/A*	94 (6)
Tumor distance to optic disc, mean mm (SD)	3.9 (3.5)
Median follow-up or follow-up for survivors, y (IQR)	7.2 (9.8)

*Abbreviations:* AJCC = American Joint Committee on Cancer; IQR = interquartile range; N/A = not applicable; SD = standard deviation.

⁎ Exact measurement of tumor diameter or thickness not available.

### Treatment failure

Of 1636 patients, 248 (15%) suffered from treatment failure. Of these 248 patients, 215 (87%) suffered from tumor recurrence, 15 (6%) from lack of regression, 7 (3%) from total retinal detachment, 4 (2%) from uncontrollable uveitis, 4 (2%) from recurring vitreous bleedings, and 3 (1%) from severe glaucoma. Of these 248 patients, 161 (65%) were treated with secondary enucleation, 83 (34%) with secondary plaque brachytherapy, and 4 (2%) with 1 or several rounds of TTT only. I-125 was selected for 11 patients (13%) and Ru-106 for 72 patients (87%) treated with secondary plaque brachytherapy. The median time elapsed between primary plaque brachytherapy and secondary treatment was 1.7 years (interquartile range, 2.1; Table 2). In Cox regression with treatment failure as a time-varying covariate, it was associated with increased risk for UM-related mortality (HR, 2.32; 95% confidence interval CI, 1.83-2.95; *P* < .001). In multivariate analysis with treatment failure as a time-varying covariate and baseline tumor diameter and thickness as covariates, treatment failure was still associated with UM-related mortality (HR, 1.98; 95% CI, 1.55-2.53; *P* < .001). Similarly, treatment failure as a time-varying covariate was associated with increased risk for all-cause mortality in univariate Cox regression (HR, 1.74; 95% CI, 1.42-2.12; *P* < .001), and the significance was retained in multivariate analysis with tumor diameter and thickness ascovariates (HR, 1.60; 95% CI, 1.30-1.96; *P* < .001).

**Table 2:** Treatment failure

Type of failure or secondary treatment	n (%)
Tumor recurrence	215 (87)
Lack of regression	15 (6)
Other*	18 (7)
**Secondary treatment**	
Enucleation	161 (65)
Plaque brachytherapy	83 (34)
Ruthenium-106	11 (13)
Iodine-125	72 (87)
TTT only	4 (2)
Median time to secondary treatment, y (IQR)	1.7 (2.1)

*Abbreviations:* IQR = interquartile range; TTT = transpupillary thermotherapy.

⁎ Total retinal detachment, uncontrollable uveitis, recurring vitreous bleedings, or severe glaucoma.

### HR for treatment failure

In univariate Cox regressions, LogMAR visual acuity, visual impairment (LogMAR ≥ 0.30), tumor diameter (continuous variable), tumor diameter >16 mm (categorical variable), tumor thickness, AJCC stage, tumor distance to optic disc ≤2 mm, absorbed dose at the sclera (continuous variable), scleral dose >400 Gy (categorical variable), and the dose rate at the tumor apex were associated with treatment failure. However, patient sex, patient age at diagnosis, ciliary body involvement, tumor distance to optic disc in mm, radioisotope (I-125 vs Ru-106), absorbed dose at the tumor apex, retinal detachment, or any of the recorded presenting symptoms were not associated with treatment failure.

The significant variables were entered into a multivariate Cox regression. As expected, only tumor distance to the optic disc ≤2 mm and scleral dose >400 Gy were independent predictors of treatment failure, as each of the other covariates were directly related to ≥1 other covariate: LogMAR visual acuity to visual impairment (LogMAR ≥ 0.30); tumor diameter, tumor diameter >16 mm, and thickness to AJCC stage; and absorbed dose at the sclera to scleral dose >400 Gy.

Lastly, visual impairment, tumor distance to optic disc of ≤2 mm, AJCC stage, and scleral dose >400 Gy were entered into a multivariate analysis. All retained their significance (Table 3).

**Table 3:** Cox regressions, hazard for treatment failure

Covariates	B	SE	Wald	*P*	Exp(B)	95% lower CI	95% upper CI
Univariate							
Patient sex, male vs female	0.14	0.13	1.24	0.27	1.15	0.90	1.48
Patient age at diagnosis*	0.01	0.05	0.01	0.92	1.00	0.91	1.09
LogMAR visual acuity†	0.37	0.16	5.63	0.02	1.44	1.07	1.95
Visual impairment‡	0.35	0.13	7.23	0.007	1.42	1.10	1.82
Tumor diameter§	0.05	0.02	7.72	0.005	1.05	1.02	1.09
Tumor diameter >16 mm	0.74	0.24	9.64	0.002	2.10	1.32	3.36
Tumor thickness§	0.07	0.03	6.26	0.01	1.07	1.01	1.13
AJCC stage║	0.25	0.08	9.68	0.002	1.28	1.10	1.50
Tumor distance to optic disc‡	−0.03	0.02	1.83	0.18	0.97	0.93	1.01
Tumor ≤2 mm from optic disc	0.32	0.14	5.35	0.021	1.37	1.05	1.80
Iodine-125 vs Ruthenium-106	−0.13	1.17	0.63	0.43	0.88	0.63	1.22
Radioactive dose at sclera¶	0.00	0.00	12.19	<0.001	1.00	1.00	1.00
Scleral dose >400 Gy	0.60	0.17	12.20	<0.001	1.83	1.30	2.57
Radioactive dose at tumor apex¶	0.00	0.01	0.17	0.68	1.00	0.99	1.01
Dose rate at tumor apex#	−0.21	0.10	4.66	0.03	0.82	0.68	0.98
Retinal detachment⁎⁎	−0.01	0.01	0.07	0.79	1.00	0.98	1.02
Shadow in visual field	0.10	0.16	0.39	0.53	1.10	0.81	1.49
Ocular pain	−0.06	0.71	0.01	0.93	0.94	0.23	3.79
Metamorphopsia	0.63	0.77	0.68	0.41	1.88	0.42	8.41
Flashes or floaters	−0.21	0.19	1.26	0.26	0.81	0.57	1.17
Multivariate							
LogMAR visual acuity†	0.22	0.25	0.74	0.39	1.24	0.76	2.04
Visual impairment‡	0.12	0.20	0.38	0.54	1.13	0.76	1.68
Tumor diameter§	−0.01	0.04	0.04	0.85	0.99	0.93	1.06
Tumor diameter >16 mm	0.31	0.34	0.84	0.36	1.37	0.70	2.69
Tumor thickness§	0.00	0.05	0.00	0.96	1.00	0.90	1.11
AJCC stage║	0.24	0.19	1.61	0.20	1.27	0.88	1.84
Tumor ≤2 mm from optic disc	0.44	0.14	9.30	0.002	1.55	1.17	2.06
Radioactive dose at sclera¶	0.00	0.00	0.57	0.45	1.00	1.00	1.00
Scleral dose >400 Gy	0.56	0.21	7.42	0.006	1.75	1.17	2.62
Dose rate at tumor apex#	0.27	0.16	3.04	0.081	1.31	0.97	1.79
Multivariate							
Visual impairment, yes vs no‡	0.28	0.14	3.92	0.048	1.32	1.00	1.75
AJCC stage║	0.20	0.09	4.39	0.036	1.22	1.01	1.47
Tumor ≤2 mm from optic disc	0.37	0.14	6.70	0.010	1.45	1.09	1.92
Scleral dose >400 Gy	0.37	0.18	4.19	0.041	1.45	1.02	2.07

*Abbreviations:* AJCC = American Joint Committee on Cancer; CI = confidence interval; SE = standard error.

⁎ Per increasing decade.

† Per increasing step on LogMAR scale, (ie, decreasing visual acuity).

‡ Defined as LogMAR ≥ 0.30.

§ Per increasing mm.

║ Per increasing stage.

¶ Per increasing Gy.

# Per increasing Gy/hour.

⁎⁎ Clearly visible upon slit-lamp biomicroscopy, not recorded if only visible on ultrasonography.

### Training cohort

Eight hundred eight patients were randomized to a training cohort for the development of the score for treatment failure risk. LogMAR visual acuity, tumor distance to optic disc, scleral dose, tumor diameter, and AJCC stage were analyzed further.

**Figure 1:**
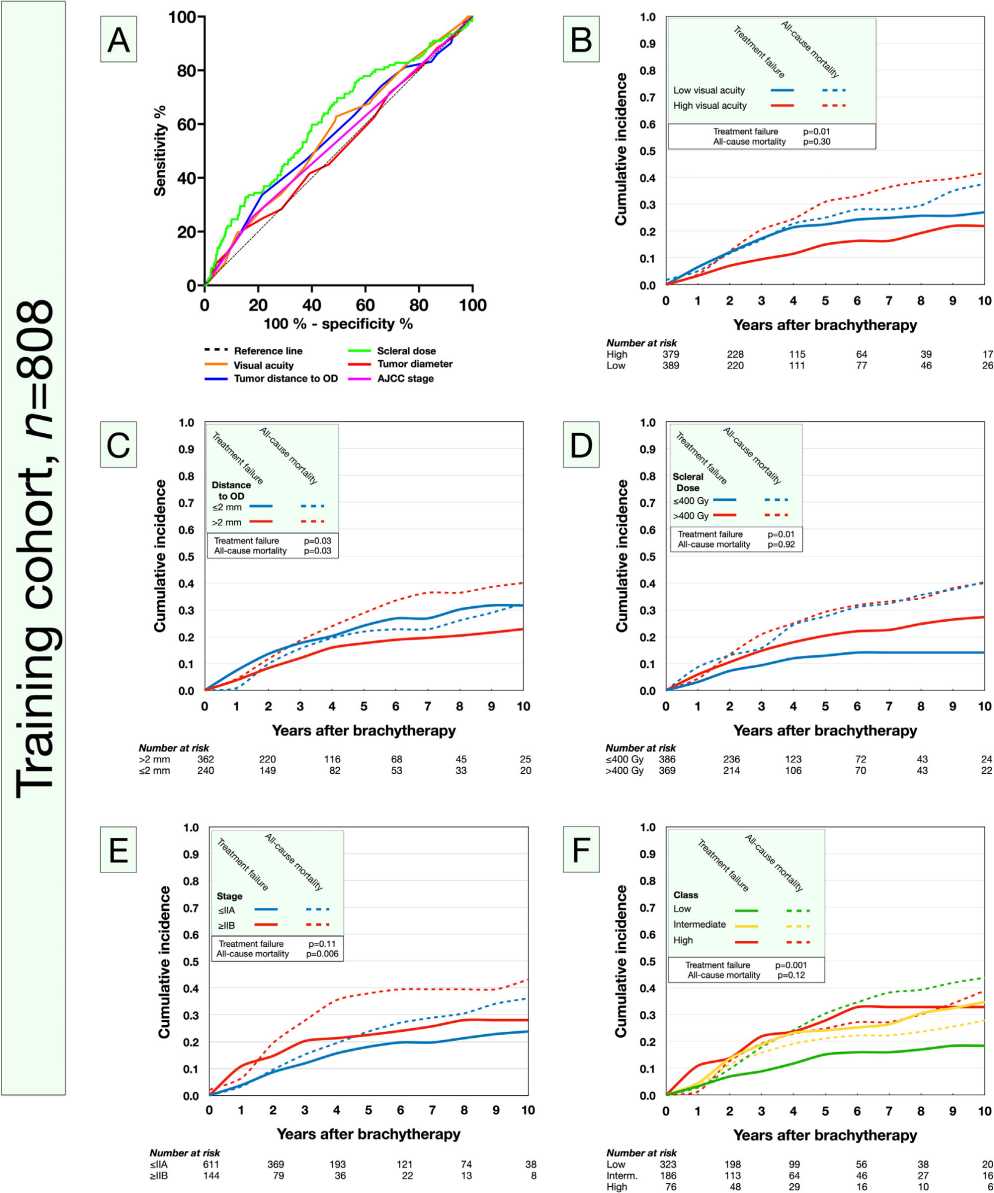
Analysis of factors related to treatment failure and development of a 3-tiered classification in the training cohort. A, Receiver operating characteristics of visual acuity (area under the curve [AUC], 0.65; 95% confidence interval [CI], 0.51-0.62), tumor distance to the optic disc (OD) (AUC, 0.56; 95% CI, 0.50-0.62), scleral dose (AUC, 0.60; 95% CI, 0.54-0.65), tumor diameter (AUC, 0.51; 95% CI, 0.46-0.57) and American Joint Committee on Cancer (AJCC) stage (AUC, 0.54; 95% CI, 0.48-0.59). B, Cumulative incidence of treatment failure and all-cause mortality for patients with high versus low visual acuity (LogMAR >0.20, corresponding to Snellen <20/32 and decimal <0.63). C, Cumulative incidences for patients with tumors that were located ≤2 versus >2 mm from the OD. D, Cumulative incidences for patients who had a scleral plaque brachytherapy dose of ≤400 versus >400 Gy. E, Cumulative incidences for patients in AJCC stage IIA or lower versus IIB or higher. No prognostic threshold could be identified for tumor diameter or AJCC stage; therefore, these factors were excluded from the final scoring system. F, The cumulative incidence of treatment failure but not all-cause mortality was higher for each tier in the final classification.

In receiver operating characteristics, maximum accuracy for visual acuity was obtained with a threshold of LogMAR > 0.20 with a sensitivity and specificity of 63 and 51% for treatment failure, respectively (area under the curve [AUC], 0.65; 95% CI, 0.51-0.62). For tumor distance to the optic disc, a threshold of ≤2 mm achieved a sensitivity and specificity of 79 and 34%, respectively (AUC, 0.56; 95% CI, 0.50-0.62). For scleral dose, a threshold of >400 Gy achieved a sensitivity and specificity of 61 and 56%, respectively (AUC, 0.60; 95% CI, 0.54-0.65). Considering the potential differences in radiobiological effects between Ru-106 and I-125, we also separated the cohorts by radioisotope: a larger AUC was obtained with I-125 (AUC, 0.76; 95% CI, 0.68-0.84) than with Ru-106 (AUC, 0.56; 95% CI, 0.52-0.61). The maximum accuracy for treatment failure was obtained at higher scleral doses with Ru-106 (sensitivity 56% and specificity 55 % at 470 Gy) than with I-125 (sensitivity 71% and specificity 73% at 370 Gy). But in Cox regression, a cutoff of >400 Gy was still significantly associated with treatment failure for both I-125 and Ru-106 in separate analyses (Ru-106: HR, 1.53, *P* = .008; I-125: HR 1.74, *P* < .001). For tumor diameter, a threshold of >15 mm achieved a sensitivity and specificity of 21 and 85%, respectively (AUC, 0.51; 95% CI, 0.46-0.57). For AJCC stage, a threshold at stage IIB or higher achieved a sensitivity and specificity of 25 and 82%, respectively (AUC, 0.54; 95% CI, 0.48-0.59; Fig. 1A).

Next, we applied these cutoffs to competing risks analyses. Patients with low visual acuity (LogMAR > 0.20), tumors ≤2 mm from the optic disc, and a scleral dose of >400 Gy had significantly higher incidences of treatment failure (Fig. 1B-D).

The incidence did not, however, differ between patients who had tumors with a diameter of >15 mm or ≤15 mm (*P* = .30). Similarly, there were no significant differences in incidence between individual AJCC stages I to IV (*P* = .36). It is of note, however, that only 19 patients were in stage IIIA and only 1 was in stage IV. Stages IIIA and IV were therefore grouped together, but differences in cumulative incidence were still not significant (*P* = .21). Next, AJCC stages were dichotomized so that stage IIA or lower was tested against stage IIB or higher. Again, cumulative incidences were not significantly different (Fig. 1E). Because of the lack of reliable thresholds for differentiating patients who suffered from treatment failure, neither tumor diameter nor AJCC stage were included in the final prognostic score.

In consideration of these results, the prognostic score was designed so that 1 point was assigned to each defined category: low visual acuity (LogMAR > 0.20), tumor ≤2 mm from the optic disc, and tumor thickness >4 for Ru-106 and >9 mm for I-125, respectively (corresponding to scleral doses of 470 and 370 Gy, respectively). Consequently, a patient could be assigned a minimum of 0 points and a maximum of 3 points. Patients in the low-risk class had ≤1 points, in the intermediate-risk class 2 points, and in the high-risk class 3 points (Table 4). The cumulative incidence of treatment failure, but not all-cause mortality, was higher for each class (*P* = .001 and *P =* .12, respectively; Fig. 1F).

**Table 4:** Treatment failure risk classification

Factor	Points
Low visual acuity (LogMAR >0.20)	1
Tumor distance to the optic disc ≤2 mm	1
Tumor thickness	
For Ruthenium-106: >4 mm	1
For Iodine-125: >9 mm	1
**Class**	
Low	≤1
Intermediate	2
High	3

### Validation cohort

**Figure 2:**
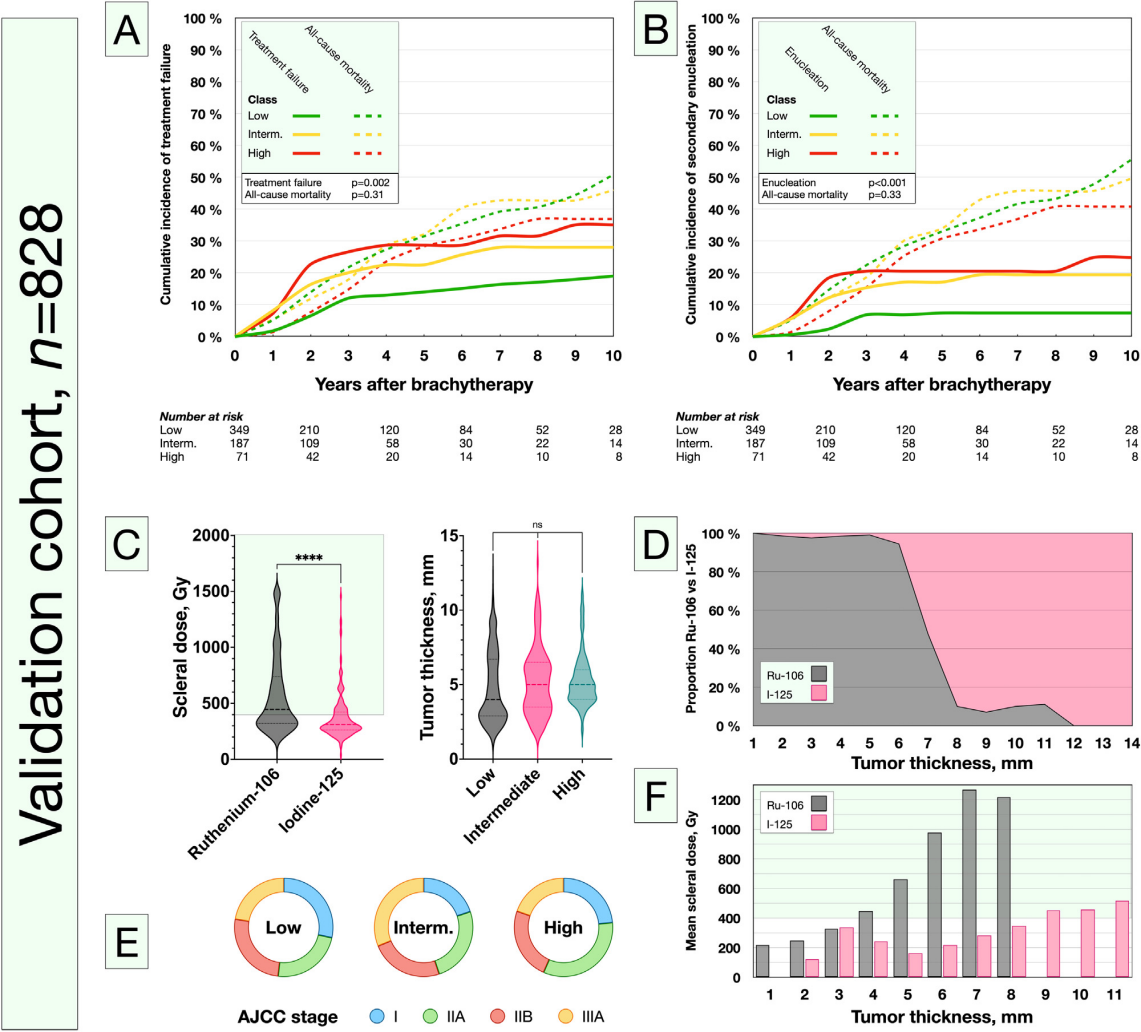
Validation cohort. A, The cumulative incidence of treatment failure was confirmed to be higher for each tier in the classification. B, Similarly, the cumulative incidence of secondary enucleation was higher for each tier in the classification. C and D, Patients treated with Ruthenium-106 had significantly higher scleral doses, but the distribution of tumor apical thickness was similar between the classes. E, The distribution of American Joint Committee on Cancer stages did not differ between the low, intermediate (interm.), and high classes (χ^2^*P* = .28). F, Scleral doses of >400 Gy were observed for ≥4 mm-thick tumors treated with Ruthenium-106 and ≥9 mm-thick tumors treated with Iodine-125. Green areas in panels C and F highlight scleral doses >400 Gy. *Abbreviation:* ns = nonsignificant. ***P < .001, **P < .01, and *P < .5.

When we tested our 3-tiered prognostic scoring system in the patients randomized to the validation cohort (n = 828), we could confirm that the low, intermediate, and high-risk classes corresponded to increasing risk for treatment failure, but not to all-cause mortality (*P* = .002 and *P* = .31, respectively; Fig. 2A). Similarly, the classes corresponded to increasing risk for secondary enucleation (*P* < .001; Fig. 2B). The cumulative 5-year incidence of treatment failure was 14% in the low-risk class, 23% in the intermediate-risk class, and 29% in the high-risk class. At 10 years, the cumulative incidence was 19, 28, and 35 %, respectively (Table E1). Patients treated with Ru-106 had significantly higher scleral doses, but the distribution of tumor apical thickness was similar between the classes (Fig. 2C, D). The distribution of AJCC stages did not differ between the low, intermediate, and high-risk classes (χ^2^ *P* = .28; Fig. 2E). As a result of the faster dose fall-off rate, scleral doses of >400 Gy was observed for >4-mm tumors treated with Ru-106, whereas the same scleral dose was reached for >9-mm tumors treated with I-125 (Fig. 2F). In Kaplan-Meier analysis, the remaining treatment failure-free proportion was lower for each tier in the classification (Log-rank *P* for trend <0.001; Fig. E1). The frequency distribution of different types of treatment failure including tumor recurrence, lack of regression, total retinal detachment, uncontrollable uveitis, recurring vitreous bleedings, and severe glaucoma differed between the classes (Table E2).

## Discussion

In this study, we demonstrate that visual impairment, tumor distance to the optic disc ≤2 mm, AJCC stage, and scleral dose >400 Gy (corresponding to >4 and >9 mm thick tumors treated with Ru-106 and I-125, respectively) are independent predictors of treatment failure in plaque brachytherapy of UM. Further, we have developed and validated a prognostic scoring system that may help clinicians and patients determine the risk for treatment failure overall as well as for secondary enucleation specifically. There is no consensus and no generally adopted limit to the acceptable risk over which primary enucleation becomes the recommendable treatment alternative. It may be suggested that a 5-year cumulative incidence of secondary enucleation of >20% is unacceptably high in a disease like UM, considering the consequences in terms of patient morbidity and, possibly, survival. Moreover, previous research has shown that patients prefer treatment alternatives with less risk for cancer recurrence over treatments with fewer side effects: Mansfield et al reported that a vast majority of patients with nonmetastatic skin melanoma preferred a theoretical intervention with 63% risk for pyrexia and 12% risk of recurrence over an intervention with 0% risk for pyrexia but with 44% risk of recurrence.^24^ This could be likened to the selection of primary plaque brachytherapy or primary enucleation, where most patients might prefer the latter even though it has greater consequences for vision and cosmetic appearance, if the risk for recurrence becomes too high with the former. It is therefore questionable if primary plaque brachytherapy is a suitable procedure for patients in the high-risk class. This corresponds to patients with low visual acuity (LogMAR > 0.20, Snellen <20/32, decimal <0.63), a tumor within 2 mm from the optic disc, and an apical thickness of >4 mm for Ru-106 and >9 mm for I-125. Ultimately, the decision on the maximal acceptable risk should be made by a well-informed patient.

The reason for the prognostic significance of a high scleral dose is likely not only related to tumor thickness. The rule of thumb is that thicker tumors will require higher scleral doses. Considering that both γ- and β-emitting radioisotopes (such as I-125 and Ru-106) are commonly used for plaque brachytherapy of UM, 6 to 7-mm-thick tumors irradiated with β-emitters will have much higher scleral doses than 8 to 12-mm-thick tumors irradiated with γ-emitters. It is therefore possible that high scleral doses are surrogates for treatment near or beyond the safe limit of the respective radioisotope. As shown here, scleral doses are higher than 400 Gy for >4-mm-thick tumors with Ru-106 and >9-mm-thick tumors with I-125 (when doses of 100 and 80 Gy are prescribed to the tumor apex, respectively). If these limits are exceeded, it seems that the risk for treatment failure increases. Regarding the theoretical differences in radiobiological effect between the 2 isotopes, a previous study found that these differences had no major clinical effect: There were no significant differences in hazard for enucleation or melanoma-related mortality in ≥5.5-mm-thick choroidal melanomas treated with Ru-106 or I-125.^21^ Similarly, in the present study, the theoretical differences in radiobiological effect between the 2 isotopes did not have a major clinical effect. Even though the highest accuracy for treatment failure was obtained at a scleral dose of 470 Gy for Ru-106 and 370 Gy for Ru-106, a threshold of >400 Gy was a predictor of treatment failure when analyzing Ru-106 and I-125 separately.

Low visual acuity is likely related to larger, more posteriorly located tumors. In turn, both factors have previously been related to tumor recurrence.^4^^,^^12^^,^^13^

Lastly, tumors within 2 mm from the optic disc are at risk of receiving suboptimal absorbed doses, as the optic nerve sheath may prevent attempts to center the plaque above the tumor.^10^ COMS, which contributes a very large share of the evidence base for current standards of plaque brachytherapy of UM, excluded lesions that had a basal diameter >16 mm and were closer than 2 mm from the optic disc.^25^ The fact that an upper threshold of 16 mm for plaque brachytherapy is now so well established in most institutions means that treatment of larger tumors are rare exceptions. They are typically only considered for treatment if the responsible ocular oncologist is convinced that treatment can be performed safely and the entire tumor irradiated. This likely selects for a group of tumors with relatively low risk for treatment failure in comparison with other large tumors. In turn, this makes the prognostic implication of a large tumor diameter weaker in statistical analyses. We did, however, demonstrate that the incidences of treatment failure and all-cause mortality are significantly higher after brachytherapy of tumors within 2 mm from the optic disc, and as shown in multivariate analysis, the increased incidence of treatment failure is independent of tumor diameter, tumor thickness, and AJCC stage. Consequently, caution is warranted when considering plaque brachytherapy of UMs in proximity of the optic disc. The exclusion of AJCC stage from the final risk classification must not be misinterpreted. It does not imply that AJCC stage is irrelevant for the risk of treatment failure. The association of cancer stage, that takes factors such as involvement of the ciliary body and the presence or absence of lymph node and distant organ metastases into account, may be less directly related to the risk of local treatment failure than the other included factors. Nevertheless, AJCC stage was an independent predictor of treatment failure in multivariate Cox regression with increasing risk for each stage, and the main reason for its exclusion from the final classification was not its prognostic irrelevance but the difficulties finding a singular clinically useful cutoff that separated low risk from high risk.

Ninety-three % of the treatment failure events were related to either tumor recurrence or lack of regression, and we believe that the factors included in the classification have a stronger relation with the risk of subthreshold radioactive doses in some areas of the tumor than to the risk of treatment toxicity. For example, the optic nerve sheath prevents optimal placing of the plaque for juxtapapillary tumors, fear of irradiating the fovea can make an ocular oncologist inclined to reduce the safety margin for a centrally located tumor, and a slightly misplaced plaque leads to larger deviations from the prescribed doses in the tumor apex of thick than in the apex of thin tumors. Most, but not all, treatment failure events occurred within the first 5 years from brachytherapy.^26^ This seems to confirm the need for very long-term follow-up of these patients. At St. Erik Eye Hospital as in most other tertiary referral centers, patients treated with plaque brachytherapy undergo ophthalmologic examination regularly (eg, every 12 months) for the remainder of their lives.

This study has several limitations. First and foremost, it is based on retrospective data from a single institution where all patients have been diagnosed and treated at the same hospital. Our treatment planning and surgical procedures will not be identical to other settings. We believe, however, that our prognostic score is well generalizable, as it was validated in an independent cohort and the factors included, save for scleral dose, have been identified as important predictors for treatment failure in cohorts from other institutions.^27^^,^^28^ However, it should not be expected that the specific incidence rates found here will be matched down to singular percentages in other samples. Moreover, patients treated during a period of 25 years have been included in this study, and it is possible that an improvement in surgical technique has affected the results. Recently, we and others have reported that UMs have become slightly smaller at diagnosis during the last several decades, which may also affect the risk for treatment failure.^3^^,^^29^ Furthermore, other statistical methods could have been used for achieving a similar guidance to other ocular oncologists. For example, some may prefer to estimate the risk for treatment failure in a nomogram rather than in a classification with fixed tiers.

## Conclusions

In conclusion, visual impairment, tumor distance ≤2 mm to the optic disc, AJCC stage, and tumor thickness are independent predictors of treatment failure in plaque brachytherapy of UM. A prognostic score was devised that identified 3 tiers of the risk for treatment failure in an independent cohort. Future studies should seek to confirm the utility of this classification in a cohort from another institution.

## Appendix Supplementary materials

Image, application 1
