# Supplementary material for: A Prognostic Score for the Prediction of Local Treatment Failure in Plaque Brachytherapy of Uveal Melanoma
Source: Adv Radiat Oncol. 2022 Dec 25;8(3):101152. doi: 10.1016/j.adro.2022.101152 (PMC9991540; doi:10.1016/j.adro.2022.101152)
Supplement: Supplementary file 1 [file mmc1.pdf]

Supplemental table 1. Cumulative incidence of treatment failure and enucleation

Treatment failure (secondary enucleation, brachytherapy, or TTT)			
**Year after diagnosis**	**Class**		
	**Low**	**Intermediate**	**High**
**1**	2%	8%	7%
**2**	7%	16%	23%
**3**	12%	20%	27%
**4**	13%	23%	29%
**5**	14%	23%	29%
**6**	15%	26%	29%
**7**	16%	28%	32%
**8**	17%	28%	32%
**9**	18%	28%	35%
**10**	19%	28%	35%
**Secondary enucleation only**			
**1**	1%	6%	6%
**2**	2%	12%	18%
**3**	7%	15%	21%
**4**	7%	17%	21%
**5**	7%	17%	21%
**6**	7%	19%	21%
**7**	7%	19%	21%
**8**	7%	19%	21%
**9**	7%	19%	25%
**10**	7%	19%	25%

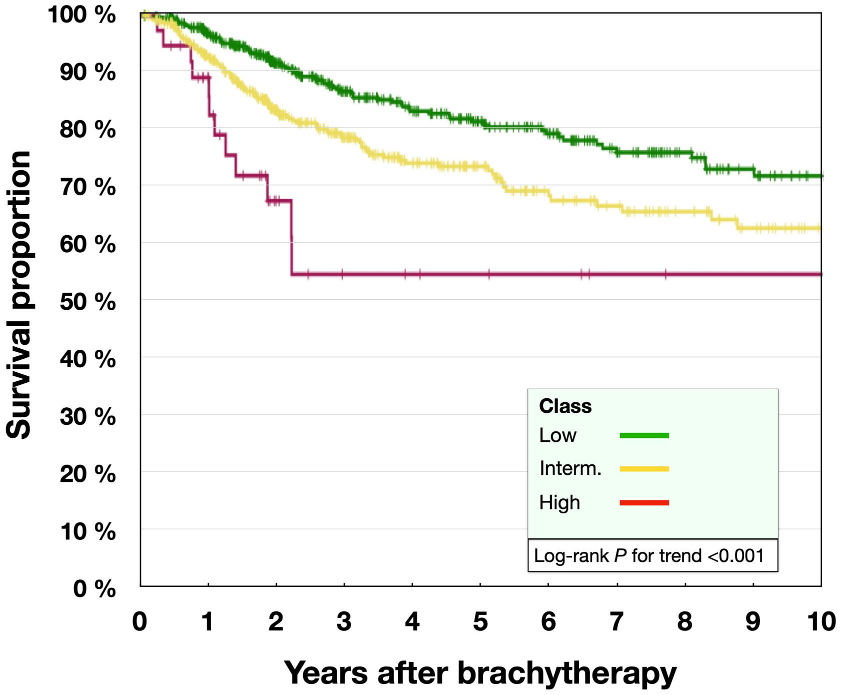

Number at risk

Low	349	210	120	84	52	28
Interm.	187	109	58	30	22	14
High	71	42	20	14	10	8

Supplemental figure. Kaplan-Meier treatment failure-free proportions of patients in the validation cohort. The remaining proportion of patients that were free from treatment failure was lower for each increasing tier in the proposed treatment failure risk classification, based on tumor thickness, visual impairment and tumor proximity to the optic disc.

Supplemental table 2. Frequency distribution of different types of treatment failures across treatment failure classes in the validation cohort.

Failure type	Class		
	**Low**	**Intermediate**	**High**
Tumor recurrence, n (%)	45 (13)	35 (19)	20 (28)
Lack of regression, n (%)	2 (1)	3 (2)	1 (1)
Total retinal detachment, n (%)	1 (<1)	2 (1)	1 (1)
Uncontrollable uveitis, n (%)	0 (0)	1 (<1)	0 (0)
Recurring vitreous bleedings, n (%)	0 (0)	1 (<1)	1 (1)
Severe glaucoma, n (%)	0 (0)	1 (<1)	1 (1)
None, n (%)	301 (86)	144 (77)	47 (66)

Chi-square *P* (5 smallest categories collapsed) <0.001
